# Association between psychological discomforts and sleep quality among people living with HIV/AIDS

**DOI:** 10.1186/s12981-023-00579-z

**Published:** 2023-11-11

**Authors:** Mohammad Ebrahimzadeh Mousavi, Safieh Mohammad Nejad, Maryam Shafaati, Rosa Mykyta-Chomsky, Samaneh Akbarpour, Fatemeh Hadavandsiri

**Affiliations:** 1 Department of Human Development and Family Sciences, University of Delaware, Newark, DE USA; 2 Department of Epidemiology and Biostatistics, School of Public Health, Tehran University of Medical Sciences, Tehran, Iran; 3 Research Center for Antibiotic Stewardship and Antimicrobial Resistance, Imam Khomeini Hospital complex, Tehran University of Medical Science, Tehran, Iran; 4 Occupational Sleep Research Center, Baharloo Hospital, Tehran University of Medical Sciences, Tehran, Iran; 5 Sleep Breathing Disorders Research Center (SBDRC), Tehran University of Medical Sciences, Tehran, Iran; 6 Department of Epidemiology, School of Public Health and Safety, Shahid Beheshti University of Medical Sciences, Tehran, Iran

**Keywords:** Depression, Anxiety, Stress, Sleep quality, HIV/AIDS

## Abstract

**Background:**

Psychological discomfort and sleep problems are considered separate disorders. Due to the high prevalence of both disorders among people living with HIV (PLWH), this study was designed to evaluate how those challenges are present among PLWH.

**Method:**

A cross-sectional study was conducted using data from a national survey of 1185 confirmed PLWH from 15 provinces in Iran from April to August 2019. Psychological discomfort and sleep quality were assessed using standardized versions of related Persian questionnaires. Logistic regression was used to assess the association between psychological discomfort and sleep quality in PLWH.

**Results:**

The overall prevalence of poor sleep quality, depression, anxiety, and stress was 47.71%, 50.95%, 44.26%, and 41.77%, respectively. The results of multivariate-adjusted logistic regression showed that each psychological discomfort covariate increased the odds of poor sleep quality. Depression by adjusting for anxiety and stress, anxiety by adjusting for depression and stress, and stress by adjusting for depression and anxiety all increased the odds of poor sleep quality.

**Conclusion:**

A high prevalence of psychological discomfort was observed in PLWH. Depression, anxiety, and stress were strongly associated with sleep quality. PLWH needed more attention and social support in order to reduce sleep and psychological issues.

**Supplementary Information:**

The online version contains supplementary material available at 10.1186/s12981-023-00579-z.

## Introduction

According to the 2018 UNAIDS Global Statistics, the number of people living with HIV/AIDS (PLWH) increased from 3.7 million to 36.9 million in just 10 years, with an estimated 1.4 to 2.4 million new HIV infections per year [1]. According to the latest estimates from UNAIDS on HIV/AIDS, there are approximately 53,000 people living with HIV in Iran until 2021, with a 95% confidence interval of 38,000 to 140,000. Of these, an estimated 17,000 are women aged 15 and over, 35,000 are men aged 15 and over, and 1,400 are children aged 0 to 14. In 2019, there were 2,500 AIDS-related deaths of all ages in Iran, with 2,000 of these deaths recorded for men between the ages of 16 and 40 [2]. PLWH reports higher rates of psychological discomfort [3], which commonly lead to poorer health outcomes in all areas of HIV care [4]. More than half of HIV-positive people struggle with psychological discomforts, such as depression, anxiety, and stress [5, 6]. The prevalence of mental disorders in the world in 2016 was estimated at 15.5%, which varies from 13 to 22% in different countries [7]. The prevalence of these disorders in Iran is reported as 21–34.2% [8]. Although a separate struggle from psychological discomfort, sleep deprivation is linked to worsening mental health outcomes and thus leads to lower treatment adherence [9]. As PLWH typically have stringent treatment regimes as well as high incidences of psychological discomfort, sleep deprivation may further exacerbate pre-existing struggles in their lives [10, 11]. These highlight the significant impact of mental health challenges on PLWH, such that the mental health challenges impact a number of HIV-related outcomes, including AIDS-related mortality for PLWH. More than one-third of PLWH report sleep disturbances as well [12]. Due to the fact that 1.2 million PLWH live in the United States, the comorbidity of mental and physical health conditions, such as depression and sleep disorders, for this population ought to be a large concern for healthcare providers. Given the well-documented strong link between sleep and depression, treatment of sleep problems in PLWH is critical to improving mental health outcomes and, thus, quality of life [10, 11, 13].

Focusing on modifiable causes of other psychological discomforts, specifically depression, anxiety, and stress, may reduce suffering and improve HIV-related outcomes through primary or secondary prevention. The present national study is the first to examine the association between psychological discomfort and sleep quality as common problems in people with HIV in Iran. It is expected that the results of this research will help to develop more comprehensive intervention methods, particularly those that increase the effectiveness of treatments and HIV prevention.

## Methods

### Study population and sample size

A total of 1,185 PLWH from April to August 2019 were enrolled in the study. To distribute the appropriate sample size across various provinces, a random selection process was employed. Three provinces were randomly chosen from each of the following regions: North-East, North-West, Central, South-East, and South-West of Iran, totaling 15 provinces. The total sample size was then divided proportionally based on the number of diagnosed patients in each province. Subsequently, a list of Voluntary Counseling and Testing Centers (VCTs) was compiled for each of the 15 provinces. From these lists, two VCTs were randomly selected in each province for data collection. Finally, within each chosen VCT, participants were selected using convenience sampling [13].

### Data collection

Interviewers who were trained and had prior experience working with HIV-positive patients were employed to collect data from confirmed HIV patients of at least 18 years of age who had been diagnosed within three months of the interview.

Data collection encompassed three parts: a physical examination, a standard questionnaire, and medical information. The physical examination involved the measurement of height and weight. For data collection, a demographic questionnaire, the Pittsburgh Sleep Quality Index (PSQI) questionnaire, and a psychological discomforts questionnaire were utilized. Additionally, medical information and disease-related data were gathered. This information included the duration of HIV diagnosis, the duration of antiretroviral therapy (ART), modes of HIV transmission, and comorbidities such as hepatitis C virus, hepatitis B virus, and tuberculosis (TB). These medical details were extracted from the electronic health records available in the Voluntary Counseling and Testing Centers (VCTs).

#### Psychological discomforts questionnaire (depression, anxiety, and stress)

The Depression, Anxiety, and Stress Questionnaire were developed to measure the psychological constructs of depression, anxiety, and stress. Its original version includes 42 questions, but later a shortened version called DASS-21 was created by Lovibond and Lovibond [14]. The shortened 21-question form was used because it is more efficient while holding on to the fundamental characteristics of the original. The validity and reliability of this questionnaire were assessed by Anthony et al. in 1998 [15]. In Iran, the validity and reliability of the questionnaire were measured by Samani and Jokar in 2008. This questionnaire consists of three scales (depression, anxiety, and stress) with questions relating to each. The scales comprised seven questions. The Cronbach’s alpha for the depression, anxiety, and stress scales was reported to be 0.81, 0.74, and 0.78, respectively [16]. Responses were rated on a four-point Likert scale ranging from 0 (not at all) to 3 (very much). The final score of each scale was obtained from the sum of the scores of the corresponding questions. Final scores categorized patients as normal, mild, moderate, severe, and extremely severe. From there, and for the final analysis, participants were categorized into two groups: normal and abnormal (all final scores other than normal—mild, moderate, severe, and extremely severe patients as defined as abnormal).

#### Pittsburgh Sleep Quality Index (PSQI) questionnaire

The Pittsburgh Sleep Quality Index (PSQI) questionnaire was used in the study as well. The validity and reliability of this questionnaire have been previously studied [17]. The Cronbach’s alpha reported for this questionnaire is 0.77. These questionnaires consist of 7 components and 19 items. Each component’s responses were rated based on a four-point Likert scale from 0 to 3, with 0 being no sleep quality issues and 3 being severe poor sleep quality. The total score of the components was obtained from the sum of the scores of the items in each component. The overall score range for the PSQI was 0–21. Good sleep quality is defined as a score of 4 or less, fairly good sleep quality as a score of 5–10, fairly bad sleep quality as a score of 11–15, and poor sleep quality as a score of 16–21 [18].

#### Ethical consideration

##### Ethical approval

was received for this study from the Tehran University of Medical Sciences that all research was performed in accordance with relevant guidelines/regulations (TUMS.FNM.REC.1399.066), and every participant provided verbal informed consent. All processes were performed in accordance with the Declaration of Helsinki.

### Statistical analysis

After removing all individuals with incomplete sleep profiles and identifying any other missing data, all other subjects missing data across any other variables (about 3.01% of the cells) were imputed by employing a single imputation method and regression model. To control sampling weight and cluster sampling, complex survey methods were applied. The weighted frequency and weighted means (and standard error) were used to describe categorical and continuous data, respectively, based on the 2021 national Iranian PLWH population for all individuals 15 years of age or older.

The p-value presented for quantitative and qualitative variables was obtained through a t-test and a chi-square test, respectively. To explore the factors associated with bad sleep quality, logistic regression was used. Sleep quality (poor sleep, good sleep) was considered the outcome variable, and psychological discomfort (depression, anxiety, and stress) was considered the independent variable.

Model 1 was run for crude odds ratios (OR). In Model 2, the exposure variable was adjusted for age and gender, and in Model 3, the same was done for the adjustment for other confounders (age, gender, BMI, CD4, Marital and Employment status, Route of transmission, HBV, HCV, Sleeping, and Mental medication). To find a clearer association between psychological discomfort and sleep, we first ran the model for depression, anxiety, and stress separately and then entered all three into the model to better adjust as a multivariate adjustment.

## Results

A total of 1185 confirmed HIV patients aged 18–78 years (weighted mean age 35.35 ± 0.06) were enrolled in the study. The overall prevalence of poor sleep quality, depression, anxiety, and stress was 47.71%, 50.95%, 44.26%, and 41.77%, respectively. The weighted mean age of patients with poor sleep quality was 35.83 ± 0.47, and the weighted mean age of patients with good sleep quality was 35.91 ± 0.43. The weighted mean CD4 score was 409.24 ± 23.10 in patients with poor sleep quality and 444.06 ± 28.8 in patients with good sleep quality. The mean BMI was almost the same in both groups (P-value = 0.128).

The prevalence of HIV-co-infection with HCV, HBV, and TB was 17.96%, 4.09%, and 7.29%, respectively, in patients with poor sleep quality and 10.89, 0.75, and 3.77 among patients without poor sleep quality, respectively. Other characteristics of the participants are shown in Table 1.

**Table 1:** Clinical and demographic characteristics between the two groups defined by sleep quality among people living with HIV/AIDS

Variables	Total (n = 1185)	Poor sleep quality (n = 480)	Good sleep quality (n = 705)	P-value
	Mean ± SE	Mean ± SE	Mean ± SE	
Age, (years)	35.35 ± 0.06	35.83 ± 0.47	35.91 ± 0.43	0.084
BMI (kg/m^2^)	23.35 ± 0.18	23.35 ± 0.24	23.34 ± 0.27	0.078
CD4 count (cells/mm^2^)	427.09 ± 18.01	409.24 ± 23.10	444.06 ± 28.08	0.053
	**Median ± IQR**	**Median ± IQR**	**Median ± IQR**	**P-value**
Duration of HIV diagnosed, months	84.5(92.23)	79.53(93.81)	87.53(91.2)	0.221
Duration of ART, months	55(60)	55(64)	54(55)	0.478
	**No (Percent)**	**No (Percent)**	**No (Percent)**	
**Sex**				
Male	717(81.27)	372(82.71)	345(79.91)	0.275
Female	468(18.72)	215(17.29)	253(20.09)	
**Marital status**				
Single	264(43.72)	137(44.49)	127(42.99)	0.367
Married	614(41.35)	292(40.25)	322(42.39)	
Divorced/widow	307(14.93)	158(15.25)	149(14.62)	
**Education**				
Under diploma	754(50.67)	383(53.07)	371(48.39)	0.179
Diploma	342(37.17)	168(37.64)	174(36.74)	
Upper diploma	89(12.15)	36(9.3)	53(14.87)	
**BMI category (kg/m**^2^)				
< 25	715(66.15)	339(63.46)	376(68.7)	0.128
25–30	301(21.18)	154(20.46)	147(21.86)	
>= 30	169(12.68)	94(16.08)	75(9.44)	
**Employment status**				
Employed	490(50.21)	227(45.14)	263(55.03)	0.064
Unemployed	695(49.79)	360(54.86)	335(44.97)	
**Route of transmission**				
Sexual contact	605(51.85)	269(49.85)	336(53.76)	P < 0.0001
Injection drug use	404(34.55)	233(40.37)	171(29)	
Blood products	10(0.6)	7(1)	3(0.22)	
Unknown	166(14.01)	78(8.78)	88(17.02)	
**Co-infection**				
HIV/HBV	34(2.38)	27(4.09)	7(0.75)	P < 0.0001
HIV/HCV	193(14.34)	118(17.96)	75(10.89)	P < 0.0001
HIV/TB	84(5.49)	51(7.29)	33(3.77)	0.034
Sleeping medication				P < 0.0001
Yes	164(13.55)	138(23.63)	26(3.96)	
No	1021(86.45)	449(76.37)	572(96.04)	
Mental medication				P < 0.0001
Yes	115(8.83)	99(16.1)	16(1.91)	
No	1070(91.17)	488(83.9)	582(98.09)	

The difference between sleep quality and depression, anxiety, and stress was statistically significant (Table 2). The weighted mean of depression, anxiety, and stress scores was higher in those with poor sleep quality than in those with good sleep quality (P < 0.0001). Only 21.97% of patients had normal scores among those who had poor sleep quality. The normal score for anxiety was 29.05%, and that for stress was 29.67% among those with poor sleep quality in both cases.

**Table 2:** Psychiatric discomforts (Depression, anxiety, and stress) and sleep quality among people living with HIV/AIDS.

Variables	Total (n = 1185)	Poor sleep quality (n = 480)	Good sleep quality (n = 705)	P-value
	Mean ± SE	Mean ± SE	Mean ± SE	
Depression score	7.33 (0.29)	10.29 (0.41)	4.50 (0.32)	P < 0.0001
Anxiety score	4.71 (0.21)	7.17 (0.34)	2.37 (0.19)	P < 0.0001
Stress score	8.33 (0.28)	11.51 (0.39)	5.3 1(0.29)	P < 0.0001
	**No (percent)**	**No (percent)**	**No (percent)**	
**Depression category**				
Normal (0–9)	524 (41.5)	141 (21.97)	383 (59.2)	P < 0.0001
Mild (10–12)	126 (11.71)	60 (7.87)	66 (15.36)	
Moderate (13–20)	222 (18.76)	142 (25.62)	80 (12.23)	
Sever (21–27)	108 (8.57)	79 (10.99)	29 (6.27)	
Extremely severe (28–42)	205 (19.91)	165 (33.54)	40 (6.95)	
**Anxiety category**				
Normal (0–6)	647(51.83)	190(29.05)	457(73.5)	P < 0.0001
Mild (7–9)	142(14.37)	78(14.59)	64(14.16)	
Moderate (10–14)	111(8.34)	78(12.51)	33(4.38)	
Sever (15–19)	86(8.08)	71(12.95)	15(3.45)	
Extremely severe (20–42)	199(17.37)	170(30.89)	29(4.51)	
**Stress category**				
Normal (0–10)	640(50.89)	196(29.67)	444(71.07)	P < 0.0001
Mild (11–18)	133(11.27)	70(11.11)	63(11.42)	
Moderate (19–26)	146(12.7)	102(16.01)	44(9.56)	
Severe (27–34)	147(12.04)	109(18.16)	38(6.21)	
Extremely severe (35–42)	119(13.1)	110(25.05)	9(1.74)	

Table 3 presents the results of multivariate-adjusted logistic regression. In this study, depression (OR = 1.77, 95% CI: 1.19–2.65), anxiety (OR = 3.34, 95% CI: 2.33–4.78), and stress (OR = 1.99, 95% CI: 1.38–2.88) increased the risk of poor sleep quality. Additionally, depression, by adjusting for anxiety and stress (OR = 5.40, 95% CI: 1.38–2.88), anxiety, by adjusting for depression and stress (OR = 6.54, 95% CI: 1.38–2.88), and stress, by adjusting for depression and anxiety (OR = 5.15, 95% CI: 1.38–2.88) increased the risk of poor sleep quality. The supplementary table presented in the study contains PSQI components.

**Table 3:** Depression, anxiety, and stress associated with sleep quality in logistic regression analysis and different models

Variables	OR^1^	CI 95% (P-value)	Multivariate adjusted OR^2^	CI 95% (P-value)
Depression^2^				
Model 1	5.63	4.37–7.25	1.82	1.28–2.57(0.001)
Model 2	5.89	4.55–7.61	1.86	1.31–2.64(P < 0.0001)
Model 3	5.29	3.98–7.03	1.89	1.29–2.76(P < 0.0001)
**Anxiety** ^**2**^				
Model 1	6.77	5.24–8.74	3.08	2.22–4.29(P < 0.0001)
Model 2	6.87	5.31–8.89	2.99	2.14–4.17(P < 0.0001)
Model 3	5.99	4.53–7.93	2.88	2.02–4.09(P < 0.0001)
**Stress** ^**2**^				
Model 1	5.75	4.47–7.39	2.16	1.55–3.02(P < 0.0001)
Model 2	6.15	4.76–7.96	2.32	1.65–3.25(P < 0.0001)
Model 3	5.13	3.89–6.76	2.04	1.42–2.92(P < 0.0001)

^1^ Model 1; Crude odds ratio, Model 2; age and sex-adjusted odds ratio, model 3 is adjusted for age, gender, BMI, CD4, Marital and Employment status, Route of transmission, HBV, HCV, Sleeping and Mental medication

^2^ Mild, moderate, severe, and extremely severe patients as defined as abnormal

^1^ Depression and anxiety and stress were entered into the model separately

^2^ Multivariate adjusted OR for depression and anxiety and stress

## Discussion

Psychological discomfort and sleep quality in PLWH in Iran, but also globally, are underrepresented in research [19]. PLWH regularly faces stigma and a lack of social support, often leading to high levels of mental illness [20]. Additionally, PLWH are more vulnerable to sleep problems [21]. Sleep quality is known as a common indicator of mental health and has been reported as especially common among people with depression (up to 90%) and other psychological discomforts (for example, 70% for those with anxiety) [22–24]. Previous research, including longitudinal studies, has implied that poor sleep quality and psychological discomfort, or otherwise poor mental health, have a complex and bidirectional relationship [11, 24, 25]. Poor sleep quality can either precede or be caused by mental problems [26]. Following the research, poorer sleep quality in PLWH will influence the quality of life and mental health [27], leading to lower medication adherence and an acceleration of HIV disease progression [28, 29].

Previous studies discussed HIV stigma as a risk factor for depression and anxiety [30]. Inversely, it is understood that HIV-related optimism leads to better psychological manifestations, thus reducing outcomes such as stress, anxiety, and depression [31]. The findings of Olagunju et al. show anxiety disorders to be five times more common in PLWH compared to the general population [32]. Poor sleep quality in the present study was 3.34-fold higher in adults with anxiety than in those without anxiety. Research has also found that greater stress is associated with greater sleep disturbances, such as insomnia [33, 34]. Stress in PLWH may be caused by stigma, joblessness, infecting others, and interpersonal relationships. Long-term stress can affect the immune system in PLWH and contribute to disease progression [35]. We found that the risk of poor sleep quality increased two-fold in individuals who have stress.

PLWH are also likely to experience greater feelings of loneliness related to increased depressive symptoms and poorer sleep quality [36]. In our study, poor sleep quality in PLWH with extremely severe depression was 31.25%. The likelihood of poor sleep quality in PLWH who also experience depression was about 77%. It has been well-documented that sleep disturbances are associated with depression, higher stress levels [9], and anxiety holding, consistent with the results of our study, which show these psychological discomforts are strongly associated with sleep in PLWH. At the same time, no significant difference was found in the mean age of individuals, gender, or BMI based on sleep quality. Studies have been done examining sleep as it relates to age in PLWH [5, 37, 38], and gender [39, 40]. There have been mixed results on both variables in past research, and this could warrant further examination. Additionally, no previous studies have been conducted examining the relationship between BMI and sleep quality for PLWH. Here as well, lies an area that ought to be studied.

Employment status is related to lower levels of educational attainment, age—younger people are more likely to be unemployed—and, relevant here, HIV-related stigma. These socio-demographic attributes all contribute to the relatively high levels of unemployment in PLWH [41]. Research shows that diagnosis of HIV infection in persons leads to loss of employment within 1 year [42]. Najafi et al., demonstrate a strong association between poor sleep quality and unemployment [5], and our results confirm this finding.

National-level data for PLWH was used for this cross-sectional study. The aim of this study was to further investigate psychological discomfort challenges and sleep quality in HIV-positive patients. Due to the nature of the study, of course, there were limitations. As in all cross-sectional studies, cross-sectional relationships cannot be determined. We did not assess the quality of life among PLWH nor their well-being in this study which could provide new insight into both mental health and sleep quality. Lastly, the ratio of male and female participants was not consistent with the official Iranian national statistics on the gender of the HIV-positive patients (81% men and 19% women); therefore, a weighting method was used to adjust this ratio based on the 2021 national Iranian PLWH population.

## Conclusion

Mental health challenges are extremely common among PLWH. Depression, anxiety, and stress, all examined in the present study, have a strong association with poor sleep quality. According to the bidirectional association between sleep and depression or anxiety, targeted management of one may improve the other. While PLWH also needs social support and other such interventions to reduce stress, depression, and anxiety and thus improve sleep quality, identifying the strong relationship between mental health and sleep quality may help inform interventions to mitigate each of the variables in tandem.

### Electronic supplementary material

Below is the link to the electronic supplementary material.

Supplementary Material 1

## Data Availability

The datasets generated and/or analyzed during the current study are not publicly available but are available from the corresponding author on reasonable request.

## Declarations

### Identifying information

According to patients with HIV/AIDS research approved by the Tehran University of Medical Sciences Ethics Committee under the code IR.TUMS.FNM.REC.1399.066, every participant provided verbal informed consent.
