# Supplementary material for: Association between psychological discomforts and sleep quality among people living with HIV/AIDS
Source: AIDS Res Ther. 2023 Nov 11;20:78. doi: 10.1186/s12981-023-00579-z (PMC10638710; doi:10.1186/s12981-023-00579-z)
Supplement: Supplementary file 1 — Supplementary Material 1 [file 12981_2023_579_MOESM1_ESM.docx]

**Supplementary table 1:** Comparison of different components of sleep quality between the different categories of psychological disorders.

| **PSQI components** | **Depressed**  **(n=669)** | **Non-Depressed**  **(n=532)** | **P-value** |
| --- | --- | --- | --- |
|  | **Mean±SE** | **Mean±SE** |  |
| **Component** 2 (Sleep latency) | 2.47±0.13 | 1.32±0.11 | P<0.0001 |
| **Component** 3 (sleep duration) | 7.36±0.11 | 7.30±0.09 | 0.764 |
| **Component** 4 (sleep efficiency) | 91.14±0.45 | 92.89±0.33 | P<0.0001 |
| **Component** 5 (sleep disturbances) | 7.04±0.34 | 3.38±0.18 | P<0.0001 |
| **Component** 7 (Daytime sleepiness) | 1.63±0.11 | 0.35±0.05 | P<0.0001 |
|  | **No (Percent)** | **No (Percent)** |  |
| **Component** 1 (quality of sleep) |  |  |  |
| very good | 60(9.98) | 153(27.79) | P<0.0001 |
| relatively good | 354(51.09) | 339(64.91) |  |
| relatively bad | 183(26.92) | 28(6.72) |  |
| very bad | 64(12) | 4(0.58) |  |
| **Component** 6 (sleep medication) |  |  |  |
| nothing | 424(66.78) | 459(84.35) | P<0.0001 |
| once a week | 58(7.55) | 35(6.91) |  |
| twice a week | 64(9.31) | 18(4.73) |  |
| Three times a week | 115(16.36) | 12(4) |  |
|  |  |  |  |
|  | **With anxiety**  **(n=651)** | **Without anxiety**  **(n=647)** | **P-value** |
| **Component** 2 (Sleep latency) | 2.72±0.14 | 1.33±0.10 | P<0.0001 |
| **Component** 3 (sleep duration) | 7.26±0.13 | 7.41±0.08 | 0.171 |
| **Component** 4 (sleep efficiency) | 90.63±0.53 | 93.00±0.27 | P<0.0001 |
| **Component** 5 (sleep disturbances) | 7.94±0.38 | 3.31±0.16 | P<0.0001 |
| **Component** 7 (Daytime sleepiness) | **1.90±0.13** | **0.37±0.04** | P<0.0001 |
|  | **No (Percent)** | **No (Percent)** |  |
| **Component** 1 (quality of sleep) |  |  |  |
| **very good** | 44(9.47) | 169(24.56) | P<0.0001 |
| **relatively good** | 275(46.29) | 418(66.49) |  |
| **relatively bad** | 159(30.22) | 52(7.86) |  |
| **very bad** | 60(14.01) | 8(1.09) |  |
| **Component** 6 (sleep medication) |  |  |  |
| nothing | 330(62.86) | 553(84.33) | P<0.0001 |
| once a week | 47(7.5) | 46(7. 1) |  |
| twice a week | 58(9.93) | 24(5.11) |  |
| Three times a week | 103(19.71) | 24(3.47) |  |
|  |  |  |  |
|  | **With stress**  **(n=545)** | **Without stress**  **(n=540)** | P-value |
|  |  |  |  |
| **Component** 2 (Sleep latency) | 2.61±0.14 | 1.40±0.11 | P<0.0001 |
| **Component** 3 (sleep duration) | 7.25±0.13 | 7.42±0.09 | 0.361 |
| **Component** 4 (sleep efficiency) | 90.70±0.51 | 92.98±0.29 | P<0.0001 |
| **Component** 5 (sleep disturbances) | 7.56±0.37 | 3.58±0.19 | P<0.0001 |
| **Component** 7 (Daytime sleepiness) | 1.81±0.12 | 0.43±0.05 | P<0.0001 |
|  | **No (Percent)** | **No (Percent)** |  |
| **Component** 1 (quality of sleep) |  |  |  |
| **very good** | 45(9.2) | 168(25.1) | P<0.0001 |
| **relatively good** | 273(47.96) | 420(65.26) |  |
| **relatively bad** | 164(28.73) | 47(8.88) |  |
| **very bad** | 63(14.11) | 5(0.75) |  |
| **Component** 6 (sleep medication) |  |  |  |
| nothing | 341(64.15) | 542(83.49) | P<0.0001 |
| once a week | 50(8.09) | 43(6.52) |  |
| twice a week | 53(9.63) | 29(5.3) |  |
| Three times a week | 101(18.13) | 26(4.69) |  |
|  |  |  |  |
